# Genomic landscape of salivary gland tumors

**DOI:** 10.18632/oncotarget.4554

**Published:** 2015-07-27

**Authors:** Shumei Kato, Sheryl K. Elkin, Maria Schwaederle, Brett N. Tomson, Teresa Helsten, Jennifer L. Carter, Razelle Kurzrock

**Affiliations:** ^1^ Department of Investigational Cancer Therapeutics, The University of Texas MD Anderson Cancer Center, Houston, TX, USA; ^2^ N-of-One, Inc., Lexington, MA, USA; ^3^ Center for Personalized Cancer Therapy and Division of Hematology and Oncology, Department of Medicine, UC San Diego, Moores Cancer Center, La Jolla, CA, USA

**Keywords:** salivary gland tumor, next-generation sequencing, genomic landscape, personalized therapy, targeted therapy

## Abstract

Effective treatment options for advanced salivary gland tumors are lacking. To better understand these tumors, we report their genomic landscape. We studied the molecular aberrations in 117 patients with salivary gland tumors that were, on physician request, tested in a Clinical Laboratory Improvement Amendments (CLIA) laboratory (Foundation Medicine, Cambridge, MA) using next-generation sequencing (182 or 236 genes), and analyzed by N-of-One, Inc. (Lexington, MA). There were 354 total aberrations, with 240 distinct aberrations identified in this patient population. Only 10 individuals (8.5%) had a molecular portfolio that was identical to any other patient (with four different portfolios amongst the ten patients).

The most common abnormalities involved the *TP53* gene (36/117 [30.8% of patients]), cyclin pathway (*CCND1*, *CDK4/6* or *CDKN2A/B*) (31/117 [26.5%]) and PI3K pathway (*PIK3CA, PIK3R1*, *PTEN* or *AKT1/3*) (28/117 [23.9%]). In multivariate analysis, statistically significant co-existing aberrations were observed as follows: *TP53* and *ERBB2* (*p* = 0.01), cyclin pathway and *MDM2* (*p* = 0.03), and PI3K pathway and *HRAS* (*p* = 0.0001). We were able to identify possible cognate targeted therapies in most of the patients (107/117 [91.5%]), including FDA-approved drugs in 80/117 [68.4%]. In conclusion, salivary gland tumors were characterized by multiple distinct aberrations that mostly differed from patient to patient. Significant associations between aberrations in *TP53* and *ERBB2*, the cyclin pathway and *MDM2*, and *HRAS* and the PI3K pathway were identified. Most patients had actionable alterations. These results provide a framework for tailored combinations of matched therapies.

## INTRODUCTION

Malignant salivary gland tumors are an uncommon subgroup of head and neck cancers [1–3]. The most frequent types of malignant salivary gland tumors according to the WHO classification [1] are mucoepidermoid carcinoma (10.0–32.7%) and adenoid cystic carcinoma (23.3–23.8%) [4, 5].

Initial standard of care therapy of localized disease is surgery and/or radiation therapy, whereas chemotherapy is typically reserved for palliative treatment of local and regional recurrence or metastatic disease [6]. Due to the rarity of the disease, data are often derived from case reports and retrospective series, rather than prospectively performed clinical trials. Thus, it has been challenging to define the role of chemotherapy in management of advanced salivary gland tumors [6]. Systemic therapies investigated in the past include cisplatin [7], paclitaxel [8], combination of cisplatin plus vinorelbine [9] and the combination of cisplatin, doxorubicin and cyclophosphamide [10]. Modest response rates can be achieved with cytotoxic chemotherapy (~4% to 27%) [7–10] and no drugs are approved by the Food and Drug Administration (FDA) specifically for salivary gland tumors.

Importantly, several possible biological targets in salivary gland tumors have been reported: c-Kit[11] (positive protein expression by immunohistochemistry but no exon 11 or 17 mutations); EGFR [12], HER2 [13, 14], androgen, estrogen and progesterone receptor protein expression by immunohistochemistry [15]; and *PIK3CA* [16] and *BRAF* mutations [16]. Interestingly, salivary duct carcinomas resemble breast cancer histologically, and about 20 to 80% of salivary duct carcinomas are HER2 positive by immunohistochemistry [13, 14]; in approximately 90% of salivary duct carcinomas, androgen receptors are positive by immunohistochemistry [17].

Although targeted therapies with imatinib [18], gefitinib [19], cetuximab [20], trastuzumab [21] and lapatinib [22] have generally had low response rates, these therapies were given to unselected patients rather than matched to individuals whose tumors harbored cognate aberrations [18–22]. However, when patients were selected for the presence of *ERBB2*/HER2 or PIK3CA aberrations and were treated with appropriate targeting agents (trastuzumab and lapatinib [23] or mTOR inhibitors [24], respectively), anecdotal remarkable responses have been described.

Given that effective treatment options are needed, further molecular understanding of salivary gland tumors is necessary. We therefore examined the genomic landscape of salivary gland malignancies, as determined by targeted next-generation sequencing (NGS). Here we report the most frequent aberrations, many of which could conceivably be actionable with targeted therapies.

## RESULTS

### Genetic aberrations in salivary gland tumors (Table 1, Figure 1 and Supplemental Table 1)

Among all salivary gland tumors (*N* = 117) that were evaluated, 41.9% (49/117) of samples were histologically diagnosed as adenoid cystic carcinoma. The second most common histology was adenocarcinoma, not otherwise specified (NOS) (39.3% [46/117]) followed by acinic cell carcinoma (6.0% [7/117]), mucoepidermoid carcinoma (4.3% [5/117]), salivary duct carcinoma (3.4% [4/117]), myoepithelial carcinoma (2.6% [3/117]) and undifferentiated carcinoma (2.6% [3/117]) (Table 1).

**Table 1:** Baseline characteristics of salivary gland tumors and frequently associated genetic aberrations

Genetic aberrations*	All *N* = 117 No. (%)	Adenoid cystic carcinoma *N* = 49 No. (%)	Adeno-carcinoma, not otherwise specified *N* = 46 No. (%)	Aciniccell carcinoma *N* = 7 No. (%)	Muco-epidermoid carcinoma *N* = 5 No. (%)	Salivary duct carcinoma *N* = 4 No. (%)	Myoepithelial carcinoma *N* = 3 No. (%)	Un-differeniated carcinoma *N* = 3 No. (%)
**TP53 (*N* = 36)**	36 (30.8)	7 (14.3)	23 (50.0)	1 (14.3)	2 (40.0)	2 (50)	0 (0)	1 (33.3)
**Cyclin pathway**^#^ **(*N* = 31)**	31 (26.5)	6 (12.2)	16 (34.8)	5 (71.4)	1 (20.0)	0 (0)	1 (33.3)	2 (66.7)
**PI3K pathway**^¥^ **(*N* = 28)**	28 (23.9)	8 (16.3)	12 (26.1)	1 (14.3)	2 (40.0)	4 (100)	1 (33.3)	0 (0)
**NOTCH1/2 (*N* = 20)**	20 (17.1)	13 (26.5)	6 (13.0)	0 (0)	0 (0)	0 (0)	1 (33.3)	0 (0)
**PIK3CA (*N* = 16)**	16 (13.7)	3 (6.1)	8 (17.4)	1 (14.3)	1 (20.0)	2 (50)	1 (33.3)	0 (0)
**KDM6A (*N* = 14)**	14 (12.0)	13 (26.5)	1 (2.2)	0 (0)	0 (0)	0 (0)	0 (0)	0 (0)
**ARID1A (*N* = 13)**	13 (11.1)	7 (14.3)	5 (10.9)	0 (0)	1 (20.0)	0 (0)	0 (0)	0 (0)
**HRAS (*N* = 13)**	13 (11.1)	2 (4.1)	5 (10.9)	1 (14.3)	1 (20.0)	2 (50)	2 (66.7)	0 (0)
**BAP1 (*N* = 10)**	10 (8.5)	4 (8.2)	3 (6.5)	1 (14.3)	2 (40.0)	0 (0)	0 (0)	0 (0)
**MDM2 (*N* = 10)**	10 (8.5)	2 (4.1)	7 (15.2)	0 (0)	0 (0)	0 (0)	0 (0)	1 (33.3)
**PTEN (*N* = 10)**	10 (8.5)	2 (4.1)	5 (10.9)	0 (0)	2 (40.0)	1 (25)	0 (0)	0 (0)
**NF1 (*N* = 8)**	8 (6.8)	0 (0)	6 (13.0)	1 (14.3)	1 (20.0)	0 (0)	0 (0)	0 (0)
**ERBB2 (*N* = 7)**	7 (6.0)	0 (0)	7 (15.2)	0 (0)	0 (0)	0 (0)	0 (0)	0 (0)

* Genetic aberrations with *N* ≥ 7 are reported.

# Cyclin pathway aberrations included *CCND1*, *CDK4/6* or *CDKN2A/B* aberrations.

¥ PI3K pathway aberrations included *PIK3CA*, *PIK3R1*, *PTEN* or *AKT1/3* aberrations.

**Figure 1:**
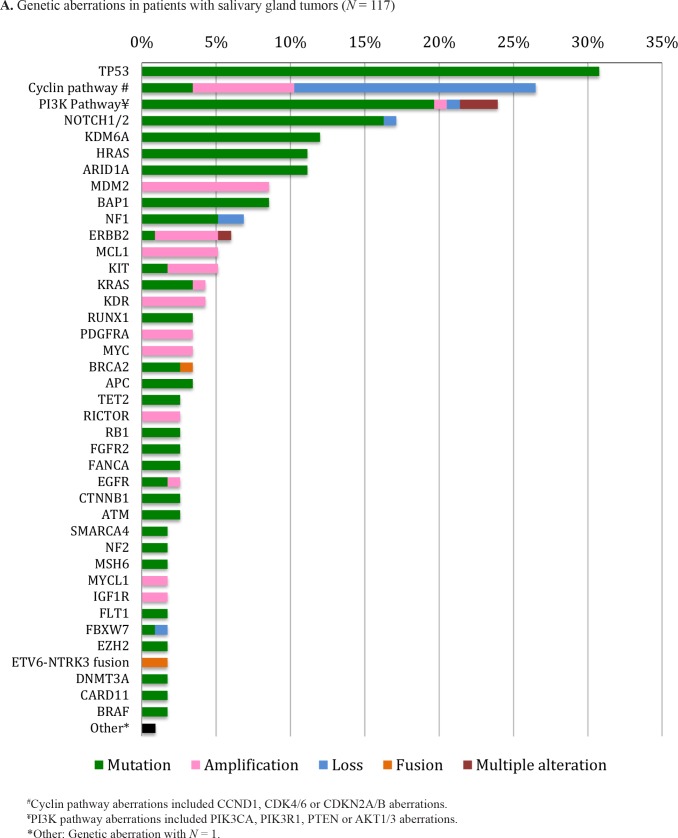

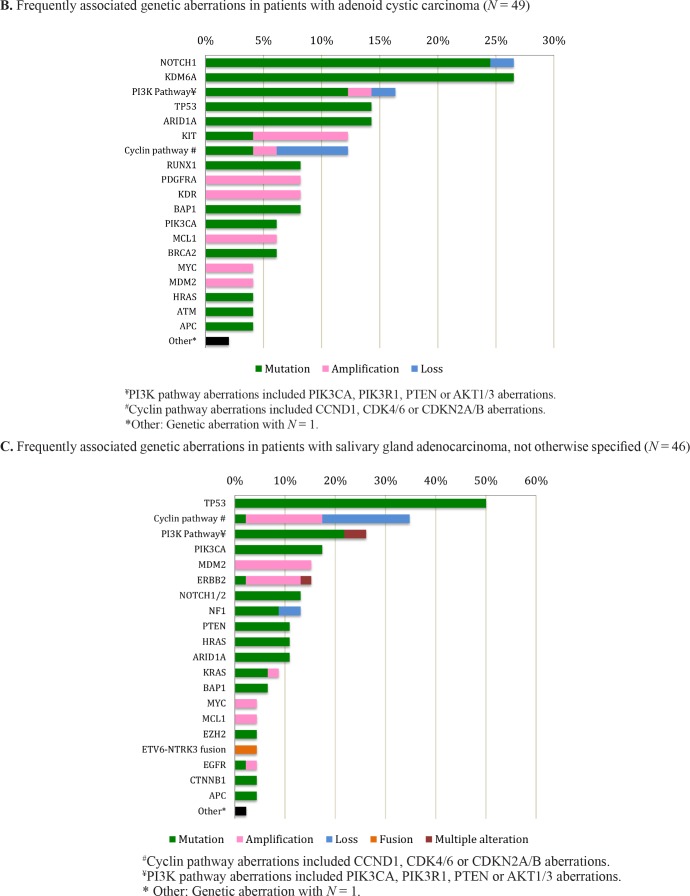
Genetic aberrations in patients with all salivary gland tumors (*N* = 117). (A) adenoid cystic carcinoma (*N* = 49) (B) and in patients with adenocarcinoma, not otherwise specified (*N* = 46) (C)

The most common aberration among all salivary gland tumors was in the *TP53* gene (36/117 patients [30.8%]), followed by anomalies in the cyclin pathway (*CCND1*, *CDK4/6* or *CDKN2A/B*) (observed in 26.5% [31/117] of all salivary gland tumors). Aberrations in the PI3K pathway (*PIK3CA, PIK3R1*, *PTEN* or *AKT*) were the third most common set of aberrations among all salivary gland tumors (28/117 [23.9%]). Anomalies in *HRAS* were seen in 11.1% (13/117) of salivary gland tumors, including 5 of 46 patients (10.9%) with adenocarcinoma, NOS (Table 1 and Figures 1A and 1C). Aberrations in *ERBB2* were found only in patients with adenocarcinoma, NOS (7/46 patients [15.2%] [two mutations and five amplifications]) (Table 1 and Figure 1C).

Molecular characteristics of patients with adenocarcinoma, NOS (*N* = 46) were similar to those of all salivary gland tumors (Table 1 and Figures 1A and 1C), likely because they were the second most common subgroup. The most common genetic aberrations among patients with adenoid cystic carcinoma (*N* = 49) were *NOTCH1/2* (26.5% [13/49]) (mainly *NOTCH1* (24.5% [12/49]) and *KDM6A* (26.5% [13/49]). Aberrations in the PI3K pathway represent the second most common genetic alterations in adenoid cystic carcinoma patients (16.3% [8/49]) (Table 1 and Figure 1B).

### Number of genetic aberrations and possible cognate targeted therapies in patients with salivary gland tumors (Figure 2, Supplemental Tables 1 and 2)

**Figure 2:**
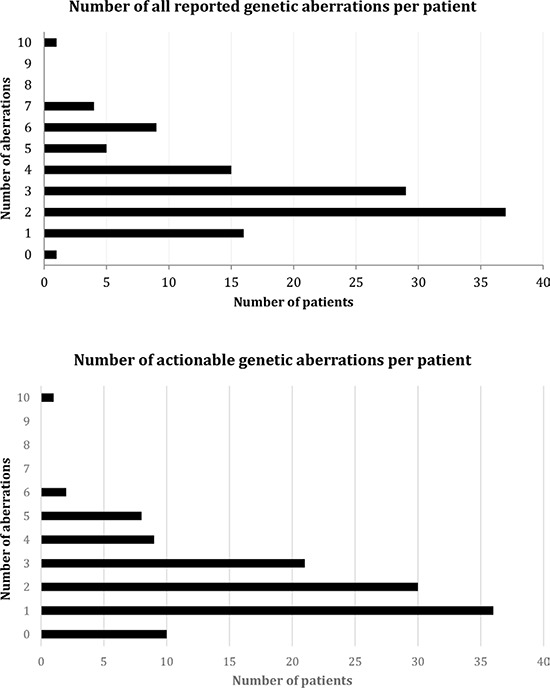
Number of reported genetic aberrations and number of theoretically actionable genetic aberrations per patient Of the 354 total aberrations (some aberrations may have been found in more than one person), 257 were actionable, with 107/117 of patients (91.5%) having a potentially actionable abnormality. Of the 240 distinct aberrations, 155 (64.6%) were potentially actionable. An aberration was considered potentially actionable if there is a drug that is approved or in clinical trials that targets that aberration with low nM IC50 or an antibody that primarily targets that abnormality.

Of the 354 total aberrations (some aberrations were identified in more than one case), 257 (72.6%) were actionable, with 107/117 patients (91.5%) having a potentially actionable abnormality. Of the 240 distinct aberrations, 155 (64.6%) were potentially actionable. Of these 155 actionable aberrations, 114 (47.5% [114/240]) were targetable by an FDA-approved drug (off label). An additional 41 (17.1% [41/240]) were targetable by an experimental drug in a clinical trial. The number of genetic aberrations reported per patient ranged from zero to ten with a median of three aberrations per patient (Figure 2). The number of genetic aberrations that were actionable ranged from zero to ten with a median of two actionable aberrations per patient (Figure 2). Of the 107 patients with at least one actionable aberration, 80 (74.8%) had an aberration targetable by an FDA-approved drug and an additional 27 (25.2%) had an aberration targetable by an investigational drug in a clinical trial (Supplemental Tables 1 and 2).

### Number of genomic aberrations and the distinctness of the profiles

As mentioned, there were 240 distinct molecular alterations. Only ten patients (8.5% [10/117]) had a molecular portfolio identical to at least one other patient (Supplemental Table 1, Case No. 13 and 1297 ([both adenocarcinoma, not otherwise specified]; No. 1523 and 1777 [both adenocarcinoma, not otherwise specified]; No. 3808, 4033, 4051 and 5681 [first three cases with acinic cell carcinoma and last past patient had adenocarcinoma, not otherwise specified]; and No. 1169 and 2807 [both adenoid cystic carcinoma] were identical). If we considered the molecular portfolio at the level of the gene, rather than the specific aberration (i.e., different abnormalities in the same gene would be considered identical), then the total number of genes altered across this cohort was 83 and, in that case, 20 patients (17.1% [20/117]) had an identical molecular portfolio to at least one other patient (Supplemental Table 1, Case No. 13 and 1297; No. 1169 and 2807; No. 1523 and 1777; No. 1939 and 6651; No. 1975 and 5730; No. 2085 and 6718; No. 2901 and 4533; No. 3808, 4033, and 5681; and No. 4332 and 4731 were identical).

### Association between *TP53* and co-existing molecular aberrations in patients with salivary gland tumors. Univariate and multivariate analysis (Table 2)

In univariate analysis, *NF1* aberrations were found to be significantly associated with *TP53* aberrations (6 of 8 patients [75.0%] with aberrant *NF1* had a *TP53* aberration; 30 of 109 patients [27.5%] with normal *NF1* had a *TP53* aberration; *p* = 0.01). Anomalies in the PI3K pathway (*PIK3CA*, *PIK3R1*, *PTEN* or *AKT1/3)* were also found to be associated with *TP53* aberrations (13 of 28 patients [46.4%] with anomalies in PI3K pathway had a *TP53* aberration; 23 of 89 patients [25.8%] with normal PI3K pathway had a *TP53* aberration; *p* = 0.06 [trend]). Interestingly, *ERBB2* aberrations were also associated with *TP53* aberrations (6 of 7 patients [85.7%] with aberrant *ERBB2* had a *TP53* aberration; 30 of 110 patients [27.3%] with normal *ERBB2* had a *TP53* aberration; *p* = 0.003).

After multivariate analysis, anomalies in the PI3K pathway and *ERBB2* aberrations were found to have an increased association with *TP53* aberrations (*p* = 0.04 and 0.01 respectively) (Table 2).

**Table 2:** Association between TP53 aberration and co-existing molecular aberrations in patients with salivary gland tumors (*N* = 117)

Patient characteristics *N* = 117	Aberrant TP53 *N* = 36 (%)	Normal TP53 *N* = 81 (%)	*p*-value* Univariate	*p*-value^¶^ Multivariate
**Co-existing aberrations**^§^				
**Cyclin pathway**^#^			0.82	
** Aberrant (*N* = 31)**	10 (32.3)	21 (67.7)		
** Wild-type (*N* = 86)**	26 (30.2)	60 (69.8)		
**PI3K pathway**^¥^			**0.06**	**0.04**
** Aberrant (*N* = 28)**	13 (46.4)	15 (53.6)		
** Wild-type (*N* = 89)**	23 (25.8)	66 (74.2)		
**NOTCH1/2**			0.11	
** Aberrant (*N* = 20)**	3 (15.0)	17 (85.0)		
** Wild-type (*N* = 97)**	33 (34.0)	64 (66.0)		
**PIK3CA**			0.25	
** Aberrant (*N* = 16)**	7 (43.8)	9 (56.3)		
** Wild-type (*N* = 101)**	29 (28.7)	72 (71.3)		
**KDM6A**			0.55	
** Aberrant (*N* = 14)**	3 (21.4)	11 (78.6)		
** Wild-type (*N* = 103)**	33 (32.0)	70 (68.0)		
**ARID1A**			0.75	
** Aberrant (*N* = 13)**	3 (23.1)	10 (76.9)		
** Wild-type (*N* = 104)**	33 (31.7)	71 (68.3)		
**HRAS**			0.22	
** Aberrant (*N* = 13)**	6 (46.2)	7 (53.8)		
** Wild-type (*N* = 104)**	30 (28.8)	74 (71.2)		
**BAP1**			0.49	
** Aberrant (*N* = 10)**	4 (40.0)	6 (60.0)		
** Wild-type (*N* = 107)**	32 (29.9)	75 (70.1)		
**MDM2**			0.17	
** Aberrant (*N* = 10)**	1 (10.0)	9 (90.0)		
** Wild-type (*N* = 107)**	35 (32.7)	72 (67.3)		
**NF1**			**0.01**	0.07
** Aberrant (*N* = 8)**	6 (75.0)	2 (25.0)		
** Wild-type (*N* = 109)**	30 (27.5)	79 (72.5)		
**ERBB2**			**0.003**	**0.01**
** Aberrant (*N* = 7)**	6 (85.7)	1 (14.3)		
** Wild-type (*N* = 110)**	30 (27.3)	80 (72.7)		

* *p*-values are from Fisher's exact test.

¶ *p* < 0.1 from univariate analysis were included in multivariate analysis using multiple logistic regression models.

§ Included characteristics with *N* ≥ 7 of genetic aberration.

# Cyclin pathway aberrations included *CCND1*, *CDK4/6* or *CDKN2A/B* aberrations.

¥ PI3K pathway aberrations included *PIK3CA*, *PIK3R1*, *PTEN* or *AKT1/3* aberrations.

### Association between aberrant cyclin pathway (*CCND1*, *CDK4/6* or *CDKN2A/B*) and co-existing molecular aberrations. Univariate and multivariate analysis (Table 3)

In univariate analysis, there was a negative association between aberrant cyclin pathway and aberrant *NOTCH1/2* (2 of 20 patients [10.0%] with aberrant *NOTCH1/2* had an abnormality in cyclin pathway; 29 of 97 patients [29.9%] with normal *NOTCH1/2* had an abnormality in cyclin pathway; *p* = 0.09 [trend]). Presence of an *MDM2* aberration was associated with abnormalities in cyclin pathway by univariate analysis (6 of 10 patients [60.0%] with aberrant *MDM2* had an abnormality in cyclin pathway; 25 of 107 patients [23.4%] with normal *MDM2* had an abnormality in cyclin pathway; *p* = 0.02).

After multivariate analysis, aberrations in *MDM2* remain positively associated with an aberrant cyclin pathway (*p* = 0.03) (Table 3).

**Table 3:** Association between aberrant cyclin pathway (*CCND1*, *CDK4/6* or *CDKN2A/B*) and co-existing molecular aberration in patients with salivary gland tumor (*N* = 117)

Patient characteristics *N* = 117	Aberrant cyclin pathway *N* = 31 (%)	Normal cyclin pathway *N* = 86 (%)	*p*-value* Univariate	*p*-value^¶^ Multivariate
**Co-existing aberrations**^§^				
**TP53**			0.82	
** Aberrant (*N* = 36)**	10 (27.8)	26 (72.2)		
** Wild-type (*N* = 81)**	21 (25.9)	60 (74.1)		
**PI3K pathway**^¥^			0.33	
** Aberrant (*N* = 28)**	5 (17.9)	23 (82.1)		
** Wild-type (*N* = 89)**	26 (29.2)	63 (70.8)		
**NOTCH1/2**			**0.09**	0.10
** Aberrant (*N* = 20)**	2 (10.0)	18 (90.0)		
** Wild-type (*N* = 97)**	29 (29.9)	68 (70.1)		
**PIK3CA**			0.55	
** Aberrant (*N* = 16)**	3 (18.8)	13 (81.3)		
** Wild-type (*N* = 101)**	28 (27.7)	73 (72.3)		
**KDM6A**			0.11	
** Aberrant (*N* = 14)**	1 (7.1)	13 (92.9)		
** Wild-type (*N* = 103)**	30 (29.1)	73 (70.9)		
**ARID1A**			0.18	
** Aberrant (*N* = 13)**	1 (7.7)	12 (92.3)		
** Wild-type (*N* = 104)**	30 (28.8)	74 (71.2)		
**HRAS**			0.18	
** Aberrant (*N* = 13)**	1 (7.7)	12 (92.3)		
** Wild-type (*N* = 104)**	30 (28.8)	74 (71.2)		
**BAP1**			0.45	
** Aberrant (*N* = 10)**	4 (40.0)	6 (60.0)		
** Wild-type (*N* = 107)**	27 (25.2)	80 (74.8)		
**MDM2**			**0.02**	**0.03**
** Aberrant (*N* = 10)**	6 (60.0)	4 (40.0)		
** Wild-type (*N* = 107)**	25 (23.4)	82 (76.6)		
**NF1**			0.68	
** Aberrant (*N* = 8)**	1 (12.5)	7 (87.5)		
** Wild-type (*N* = 109)**	30 (27.5)	79 (72.5)		
**ERBB2**			1.00	
** Aberrant (*N* = 7)**	2 (28.6)	5 (71.4)		
** Wild-type (*N* = 110)**	29 (26.4)	81 (73.6)		

* *p*-values are from Fisher's exact test.

¶ *p* < 0.1 from univariate analysis were included in multivariate analysis using multiple logistic regression models.

§ Included characteristics with *N* ≥ 7 of genetic aberration.

¥ PI3K pathway aberrations included *PIK3CA*, *PIK3R1*, *PTEN* or *AKT1/3* aberrations.

### Association between PI3K pathway abnormalities (*PIK3CA, PTEN, AKT1/3* aberrations) and co-existing molecular aberrations. Univariate and multivariate analysis (Table 4)

In univariate analysis, aberrations in *HRAS* were associated with PI3K pathway abnormalities (10 of 13 patients [76.9%] with aberrant *HRAS* had a PI3K pathway abnormality; 18 of 104 patients [17.3%] with normal *HRAS* had a PI3K pathway abnormality; *p* = < 0.0001).

A trend toward an association between aberrations in *NF1* and PI3K pathway abnormalities was noted (4 of 8 patients [50.0%] with aberrant *NF1* had an abnormality in the PI3K pathway; 24 of 109 patients [22.0%] with normal *NF1* had an abnormality in PI3K pathway; *p* = 0.09 [trend]). As mentioned earlier (Table 2), there was a positive correlation between PI3K pathway abnormalities and aberrant *TP53* (13 of 36 patients [36.1%] with aberrant *TP53* had an abnormality in the PI3K pathway; 15 of 81 patients [18.5%] with normal *TP53* had an abnormality in the PI3K pathway; *p* = 0.06 [trend]).

The correlation between aberrant *TP53* and PI3K pathway was no longer seen after multivariate analysis (Tables 2 and 4). However, the association between aberrant *HRAS* and PI3K pathway abnormalities remained statistically significant (*p* = 0.0001) (Table 4).

**Table 4:** Association between PI3K pathway abnormalities (*PIK3, PIK3R1, PTEN*, or *AKT1/3* aberrations) and co-existing molecular aberration in patients with salivary gland tumor (*N* = 117)

Patient characteristics *N* = 117	Aberrant PI3K pathway *N* = 28 (%)	Normal PI3K pathway *N* = 89 (%)	*p*-value* Univariate	*p*-value^¶^ Multivariate
**Co-existing aberrations**^§^				
**TP53**			**0.06**	0.22
** Aberrant (*N* = 36)**	13 (36.1)	23 (63.9)		
** Wild-type (*N* = 81)**	15 (18.5)	66 (81.5)		
**Cyclin pathway^#^**			0.33	
** Aberrant (*N* = 31)**	5 (16.1)	26 (83.9)		
** Wild-type (*N* = 86)**	23 (26.7)	63 (73.3)		
**NOTCH1/2**			0.15	
** Aberrant (*N* = 20)**	2 (10.0)	18 (90.0)		
** Wild-type (*N* = 97)**	26 (26.8)	71 (73.2)		
**KDM6A**			0.51	
** Aberrant (*N* = 14)**	2 (14.3)	12 (85.7)		
** Wild-type (*N* = 103)**	26 (25.2)	77 (74.8)		
**ARID1A**			0.19	
** Aberrant (*N* = 13)**	1 (7.7)	12 (92.3)		
** Wild-type (*N* = 104)**	27 (26.0)	77 (74.0)		
**HRAS**			**<0.0001**	**0.0001**
** Aberrant (*N* = 13)**	10 (76.9)	3 (23.1)		
** Wild-type (*N* = 104)**	18 (17.3)	86 (82.7)		
**BAP1**			0.70	
** Aberrant (*N* = 10)**	3 (30.0)	7 (70.0)		
** Wild-type (*N* = 107)**	25 (23.4)	82 (76.6)		
**MDM2**			1.00	
** Aberrant (*N* = 10)**	2 (20.0)	8 (80.0)		
** Wild-type (*N* = 107)**	26 (24.3)	81 (75.7)		
**NF1**			**0.09**	0.16
** Aberrant (*N* = 8)**	4 (50.0)	4 (50.0)		
** Wild-type (*N* = 109)**	24 (22.0)	85 (78.0)		
**ERBB2**			1.00	
** Aberrant (*N* = 7)**	1 (14.3)	6 (85.7)		
** Wild-type (*N* = 110)**	27 (24.5)	83 (75.5)		

* *p*-values are from Fisher's exact test.

¶ *p* < 0.1 from univariate analysis were included in multivariate analysis using multiple logistic regression models.

§ Included characteristics with *N* ≥ 7 of genetic aberration.

# Cyclin pathway aberrations included *CCND1*, *CDK4/6* or *CDKN2A/B* aberrations.

## DISCUSSION

Malignant salivary gland tumors are an uncommon type of cancer of the head and neck [1, 2]. In general, salivary gland tumors have shown low response rates to chemotherapies [7–10] or to molecularly targeted therapies that are administered without molecular matching [11–13, 15, 16, 18–22]. Thus, therapeutic options for salivary gland tumors are limited. However, anecdotal reports have described remarkable responses in salivary tumors when genetic aberrations and therapies were matched: trastuzumab and lapatinib for Her2-aberrant salivary tumors [23] or mTOR inhibitors for *PIK3CA*-aberrant neoplasms [24]. We therefore investigated the genomic landscape of salivary gland tumors by targeted next-generation sequencing.

In our current study of 117 patients suffering from salivary gland tumors, the most common histological diagnosis was adenoid cystic carcinoma (41.9% [49/117]). The second most common histology was adenocarcinoma, NOS (39.3% [46/117]) followed by acinic cell carcinoma (6.0% [7/117]) and mucoepidermoid carcinoma (4.3% [5/117]) (Table 1).

It is unclear why the frequency of histological subtypes of salivary gland tumors seen in this study is different from the previous literature; mucoepidermoid carcinoma and adenoid cystic carcinoma are the two most common subtypes [4, 5] and adenocarcinoma, NOS has been reported to be relatively low in frequency (1.8 - 3.3%) [4, 5]. However it is plausible that aggressive histological subtypes of malignant salivary gland tumors are more frequently referred for next-generation sequencing in order to pursue possible treatment options. Indeed, Wahlberg *et al* reported that patients with adenocarcinoma, NOS had worse 10-year survival compared to mucoepidermoid and adenoid cystic carcinoma (10-year survival 55%, 80%, and 74% respectively) [3].

The most common genetic aberration among 117 patients with salivary gland tumors was *TP53* mutation (36/117 [30.8%]) (Table 1, Figure 1A and Supplemental Table 1). Our current study is in agreement with previous reports demonstrating that 22–60% of salivary gland tumors harbor a *TP53* mutation [25–27]. Although little is known about the role of *TP53* in the pathogenesis of salivary gland tumors, it has been suggested that alteration in this gene is involved in later stages of tumor progression [28]. Of interest, Said *et al* showed that bevacizumab-containing regimens were associated with longer progression-free survival (PFS) when compared to non-bevacizumab-containing regimens in patients with *TP53*-mutated advanced solid tumors (median 11.0 versus 4.0 months; PFS was not increased in patients with wild-type *TP53* on bevacizumab-based treatment [median = 5 months, *p* < 0.0001]) [29]. These results require validation in a prospective study. Of interest, in our study, aberrations in *ERBB2* were associated with *TP53* anomalies after multivariate analysis (*p* = 0.01) (Table 2). Anomalies in *ERBB2* may be targeted with lapatinib or trastuzumab [23, 30].

The second most common aberrations involved the cyclin pathway (*CCND1, CDK4/6* or *CDKN2A/B*), which was abnormal in 26.5% of patients (31/117) with salivary gland tumors. Aberrations in the cyclin D-cyclin-dependent kinase pathway that regulates the cell cycle restriction point is a common feature of human cancer, contributing to tumor proliferation, genomic instability and chromosomal instability [31–33]. This pathway can be altered through multiple mechanisms including increased signaling through *CDK4* and *CDK6* amplification, overexpression of cyclin D1, and loss of inhibitors including *CDKN2A* (p16) and/or *CDKN2B* (p15) [34–37]. Mutation or loss of *RB1* (Rb; retinoblastoma) also alters this pathway, but renders tumors resistant to CDK inhibitors. Only three patients in our series had *RB1* mutations. According to Etges *et al* [38], malignant salivary gland tumors expressed the cyclin pathway differently from normal salivary gland when assessed by immunohistochemistry. They reported that expression of cyclin D1, CDK4 and CDKN2A were significantly higher in malignant salivary gland tumors (including adenoid cystic carcinoma and mucoepidermoid carcinoma) when compared to normal salivary gland. Meanwhile protein expression level of Rb was lower in malignant salivary gland tumors when compared to normal salivary gland [38]. Although it is unclear why CDKN2A protein expression was higher in malignant salivary gland tumors in this study, it is possible that the cyclin pathway is involved in tumorigenesis of salivary gland cancers. Regarding therapeutic implications, the cyclin pathway is possibly targetable with CDK4/6 inhibitors such as with palbociclib [33] and further investigation is warranted. Of interest, *MDM2* amplifications were more likely to be associated with abnormalities in the cyclin pathway (6/10 [60.0%] versus 25/107 [23.4%]; *p* = 0.03 after multivariate analysis) (Table 3). Similarly, Moller *et al* reported that five out of seven patients with diffuse large B cell lymphomas had co-aberrations in *MDM2* (amplification) and *CDKN2A* (deletion), when assessed by immunohistochemistry and polymerase chain reaction respectively [39]. Since MDM2 is negatively regulated by *CDKN2A*, *MDM2* amplification and aberrations in the cyclin pathway will likely lead to suppression of *TP53* (*TP53* is negatively regulated by *MDM2*) [40]. Thus, for patients with co-aberrations in *MDM2* and the CDK pathway, inhibition of both MDM2 [41] and the cyclin pathway [33] may be required to effectively target this pathway.

Importantly, aberrations in the PI3K pathway (*PIK3CA, PIK3R1*, *PTEN* or *AKT*) were commonly seen in salivary gland tumors (28/117 [23.9%]). Of interest, there was a statistically significant association between aberrant *HRAS* and PI3K pathway abnormalities (10/13 [76.9%] versus 18/104 [17.3%]; *p* = 0.0001 after multivariate analysis, Table 4). PI3K and RAS are key regulators of cell motility and chemotaxis and they influence each other's activities by direct and indirect feedback processes [42]. Janku *et al* have also previously shown that MAP (mitogen-activated protein) kinase-related genes are more frequently aberrant in the presence *PIK3CA* mutations than when *PIK3CA* is normal [43]. Since mutation in *HRAS* is also capable of activating the MAP kinase signaling pathway, merely blocking the PI3K pathway will likely not be sufficient. Although the role of *PIK3CA* mutation as a predictive biomarker for everolimus or PI3K inhibitor response is controversial [44], the PI3K pathway is potentially targetable with the mTOR inhibitor everolimus [45]. Adding MEK inhibitors such as trametanib [46] in order to block signals downstream of HRAS may be required in patients who have anomalies in the PI3K pathway.

Our results in adenoid cystic carcinoma (Figure 1B) are in agreement with previously reported literature where several aberrations including *NOTCH1/2* (3/24 [12.5%]), *KDM6A* (2/24 [8.3%]), *CDKN2A* (1/24 [4.2%]) and *PIK3CA* (1/24 [4.2%]) were identified by whole exome sequencing of 24 patients with adenoid cystic carcinoma [47]. Both studies had small numbers of patients, reflecting the rarity of this tumor.

Interestingly, 107/117 patients (91.5%) had potentially actionable aberrations (Supplemental Table 1). The number of actionable genes affected per patient ranged between zero and ten, with a median of two per patient (Figure 2). Of the 240 distinct aberrations, 155 (64.6%) were potentially actionable. Of these 155 actionable aberrations, 114 were targetable by an FDA-approved drug (off label) (representing 47.5% [114/240] of all distinct alterations). An additional 41 (17.1% [41/240] of all distinct alterations) were targetable by an experimental drug in a clinical trial. (Figure 2, Supplemental Table 1 and 2). Overall, 91.5% (107/117) of patients had at least one potentially actionable anomaly. As there are no FDA-approved targeted therapies for salivary gland tumors and most conventional chemotherapy has been shown to be associated with poor clinical outcomes (median overall survival [OS]: 4 to 21 months in the advanced setting) [7–10], targeted therapies based on molecular profiling merit investigation [48].

Our current study has some limitations. First, it was performed retrospectively with a relatively limited number of patients, especially in certain subgroups of salivary gland tumors. The small numbers preclude definitive statistical conclusions in some areas. Second, multiple comparisons could result in overcalling the implications of positive *p* values. Third, we included heterogeneous salivary gland tumors. Fourth, molecular analysis was performed on archival tumor tissue, which was obtained at a different time points in relationship to the clinical history. Lastly, lumping the common pathway abnormalities together such as cyclin pathway (*CCND1*, *CDK4/6* or *CDKN2A/B*) and PI3K pathway (*PIK3CA*, *PIK3R1*, *PTEN* or *AKT1/3*) aberrations may be misleading since different mutations can lead to diverse functional consequences. However, despite these limitations, this genomic characterization of salivary gland tumors has uncovered some interesting and clinically relevant results.

In conclusion, our 117 patients with salivary gland malignancies harbored 354 alterations (median = 3 per patient), 240 of which were distinct aberrations. The most common aberrations in patients with salivary gland tumors were in the *TP53* gene, followed by alterations in the cyclin pathway and the PI3K pathway (*PIK3CA, PIK3R1*, *PTEN* or *AKT*) (Table 1 and Figure 1A). Interestingly, in multivariate analysis, there was a significant independent association between alterations in the *TP53* and *ERBB2* genes (*p* = 0.01) (Table 2), cyclin pathway and the *MDM2* gene (*p* = 0.03) (Table 3), and between *HRAS* and the PI3K pathway (*p* = 0.0001) (Table 4), suggesting that dual targeting with cognate inhibitors may be necessary to overcome resistance. The vast majority of patients (91.5%) had at least one aberration that was potentially targetable by an FDA-approved drug or an investigational agent in a clinical trial. Indeed, of the 240 distinct aberrations, 155 (64.6%) were potentially actionable. These observations suggest that matching patients with appropriately targeted agents is feasible and warrants study. However, only 10 of 117 patients (8.5%) had a molecular portfolio identical to at least one other patient. The latter results are similar to those that we reported in metastatic breast cancer, where we recently described 131 distinct aberrations in 57 patients with no two patients having the same molecular portfolio [49–51]. Taken together, these observations suggest that customized combination therapy may have the potential to provide significant benefit for these patients.

## MATERIALS AND METHODS

### Patients

We investigated the genomic aberration status of patients with salivary gland tumors referred to Foundation Medicine (Cambridge, MA) for NGS from October 2011 to November 2013 (*N* = 117). We retrospectively reviewed the histological types of salivary gland tumors and associated genetic aberrations. Here, we report on the prevalence and frequencies of these aberrations in salivary gland tumors.

### Tissue samples and mutational analysis

Available tissues from diagnostic and therapeutic procedures were used to assess molecular aberrations. Samples from formalin-fixed paraffin-embedded tissue were sent for targeted NGS at Foundation Medicine (Cambridge, MA). The test sequences the entire coding sequence of 182, or more recently 236, cancer-related genes plus 47 introns from 19 genes often rearranged or altered in cancer to an average depth-of-coverage of greater than 250X (http://foundationone.com/docs/FoundationOne_tech-info-and-overview.pdf).

This method of sequencing allows for detection of copy number alterations, gene rearrangements, and somatic mutations with 99% specificity and >99% sensitivity for base substitutions at ≥5 mutant allele frequency and >95% sensitivity for copy number alterations. Foundation Medicine uses a threshold of ≥8 copies for gene amplification. The submitting physicians provided specification of tumor types. Next-generation sequencing data were collected and interpreted by N-of-One, Inc. (Lexington, MA; http://www.n-of-one.com). Data was analyzed in accordance with UCSD IRB guidelines. For the purpose of our analysis, “phosphoinositide 3-kinase (PI3K) pathway” alterations included alterations of *PIK3CA, PIK3RI, PTEN*, or *AKT1/3*. Similarly, “cyclin pathway” alterations included *CCND1, CDK4/6*, or *CDKN2A/B* alterations. We have evaluated if certain genomic aberrations were actionable or not based on the availability of drug that is approved or in clinical trials that targets that aberration with low nM IC50 or an antibody that primarily targets that abnormality.

### Endpoints and statistical methods

Descriptive statistics were used to summarize the baseline patient characteristics. The Fisher's exact test was used to assess the association between categorical variables in univariate analysis. Multiple logistic regression models were used for multivariable analysis. All tests were 2-sided. Statistical analyses were carried out using GraphPad Prism version 6.0 (San Diego, CA, USA) and SPSS version 22.0 (Chicago, IL, USA).

## SUPPLEMENTARY TABLES




