# Supplementary material for: Genomic landscape of salivary gland tumors
Source: Oncotarget. 2015 Jul 27;6(28):25631–45. doi: 10.18632/oncotarget.4554 (PMC4694855; doi:10.18632/oncotarget.4554)
Supplement: Supplementary file 1 [file oncotarget-06-25631-s001.pdf]

SUPPLEMENTARY TABLES

Supplementary Table S1: Patient characteristics and molecular profiling of 117 salivary gland tumors and examples of possible cognate targeted therapies.

Supplementary Table S2: Summary of examples of possible cognate targeted therapies.

Therapies	Cognate Targets
Bevacizumab (pilot retrospective data)	*TP53* mutations [S6]
Crizotinib, TRK inhibitor LOXO-101*	*ETV6-NTRK3* [S1]
MDM2 inhibitor *	*MDM2* [S3]
Palbociclib [S2] *	*CDKN2A/B* loss, *CDK4/6* amplification, *CCND1* amplification, *CCND3* amplification
Everolimus	*PIK3CA*, *PIK3R1*, *PTEN* mutation [S4, 5], *NF2* mutation [S13], *NF1* mutation [S16, 17], *AKT1/3* mutation [S4, 5], *RICTOR* amplification [S26]
Trametinib	*KRAS* mutation [S7], *HRAS* mutation [S7], *NF1* mutation [S18]
CTNNB1 pathway inhibitors in clinical trials *[S21]	*CTNNB1* mutation
Sorafenib	*MCL1* mutation [S19]
Pazopanib, lenvatinib* or dovitinib* [S23]	*FGFR* aberration
Sunitinib [S25] or sorafenib [S10]	*FLT1* aberration
Olaparib	*BRCA2* mutation [S22], *ATM* mutation [S28]
IGF1R inhibitor*	*IGF1R* aberration [S24]
Erlotinib or cetuximab	*EGFR* aberration [S20]
Cabozantinib	*RET* aberration [S29]
Imatinib [S8], dasatinib, sunitinib [S9], sorafenib [S10] and regorafenib [S11]	*KIT* aberration
Imatinib [S8], dasatinib, sunitinib [S9], sorafenib [S10] and regorafenib [S11]	*PDGFR* aberration
Carfilzomib	*CARD11*aberration [S32, 33]
Vemurafenib and dabrafenib	*BRAF*aberration [S34]
Ruxolitinib	*JAK2* aberration [S35]
Trastuzumab or lapatinib	*ERBB2* aberration [S30]
Crizotinib	*ALK* aberration [S38]
Vismodegib [S15]	*PTCH1*aberration
Gamma-secretase inhibitor* [S14]	*NOTCH* aberration
Dacomitinib* [S27]	*ERBB4* aberration
Aurora Kinase A or B inhibitors*[S31]	*AURKA, AURKB* aberration
Cabozantinib [S12]	*KDR* aberration

*Therapies currently in clinical trial.
All other drugs are FDA approved for other types of cancer treatment.

SUPPLEMENTARY REFERENCES

1. Taipale M, Krykbaeva I, Whitesell L, Santagata S, Zhang J, Liu Q, Gray NS and Lindquist S. Chaperones as thermodynamic sensors of drug-target interactions reveal kinase inhibitor specificities in living cells. Nat Biotechnol. 2013; 31:630–637.
2. Rocca A, Farolfi A, Bravaccini S, Schirone A and Amadori D. Palbociclib (PD 0332991) : targeting the cell cycle machinery in breast cancer. Expert Opin Pharmacother. 2014; 15:407–420.
3. Khoo KH, Verma CS and Lane DP. Drugging the p53 pathway: understanding the route to clinical efficacy. Nat Rev Drug Discov. 2014; 13:217–236.
4. Janku F, Wheler JJ, Westin SN, Moulder SL, Naing A, Tsimberidou AM, Fu S, Falchook GS, Hong DS, Garrido-Laguna I, Luthra R, Lee JJ, Lu KH and Kurzrock R. PI3K/AKT/mTOR inhibitors in patients with breast and gynecologic malignancies harboring PIK3CA mutations. J Clin Oncol. 2012; 30:777–782.
5. Janku F, Hong DS, Fu S, Piha-Paul SA, Naing A, Falchook GS, Tsimberidou AM, Stepanek VM, Moulder SL, Lee JJ, Luthra R, Zinner RG, Broaddus RR, Wheler JJ and Kurzrock R. Assessing PIK3CA and PTEN in early-phase trials with PI3K/AKT/mTOR inhibitors. Cell Rep. 2014; 6:377–387.
6. Said R, Hong DS, Warneke CL, Lee JJ, Wheler JJ, Janku F, Naing A, Falchook GS, Fu S, Piha-Paul S, Tsimberidou AM and Kurzrock R. P53 mutations in advanced cancers: clinical characteristics, outcomes, and correlation between progression-free survival and bevacizumab-containing therapy. Oncotarget. 2013; 4:705–714.
7. Infante JR, Fecher LA, Falchook GS, Nallapareddy S, Gordon MS, Becerra C, DeMarini DJ, Cox DS, Xu Y, Morris SR, Peddareddigari VG, Le NT, Hart L, Bendell JC, Eckhardt G, Kurzrock R, et al. Safety, pharmacokinetic, pharmacodynamic, and efficacy data for the oral MEK inhibitor trametinib: a phase 1 dose-escalation trial. Lancet Oncol. 2012; 13:773–781.
8. Corless CL, Schroeder A, Griffith D, Town A, McGreevey L, Harrell P, Shiraga S, Bainbridge T, Morich J and Heinrich MC. PDGFRA mutations in gastrointestinal stromal tumors: frequency, spectrum and in vitro sensitivity to imatinib. J Clin Oncol. 2005; 23:5357–5364.
9. Dewaele B, Wasag B, Cools J, Sciot R, Prenen H, Vandenberghe P, Wozniak A, Schoffski P, Marynen P and Debiec-Rychter M. Activity of dasatinib, a dual SRC/ABL kinase inhibitor, and IPI-504, a heat shock protein 90 inhibitor, against gastrointestinal stromal tumor-associated PDGFRAD842V mutation. Clin Cancer Res. 2008; 14:5749–5758.
10. Wilhelm SM, Adnane L, Newell P, Villanueva A, Llovet JM and Lynch M. Preclinical overview of sorafenib, a multikinase inhibitor that targets both Raf and VEGF and PDGF receptor tyrosine kinase signaling. Mol Cancer Ther. 2008; 7:3129–3140.
11. George S, Wang Q, Heinrich MC, Corless CL, Zhu M, Butrynski JE, Morgan JA, Wagner AJ, Choy E, Tap WD, Yap JT, Van den Abbeele AD, Manola JB, Solomon SM, Fletcher JA, von Mehren M, et al. Efficacy and safety of regorafenib in patients with metastatic and/or unresectable GI stromal tumor after failure of imatinib and sunitinib: a multicenter phase II trial. J Clin Oncol. 2012; 30:2401–2407.
12. Yakes FM, Chen J, Tan J, Yamaguchi K, Shi Y, Yu P, Qian F, Chu F, Bentzien F, Cancilla B, Orf J, You A, Laird AD, Engst S, Lee L, Lesch J, et al. Cabozantinib (XL184), a novel MET and VEGFR2 inhibitor, simultaneously suppresses metastasis, angiogenesis, and tumor growth. Mol Cancer Ther. 2011; 10:2298–2308.
13. Schroeder RD, Angelo LS and Kurzrock R. NF2/merlin in hereditary neurofibromatosis 2 versus cancer: biologic mechanisms and clinical associations. Oncotarget. 2014; 5:67–77.
14. Messersmith WA, Shapiro GI, Cleary JM, Jimeno A, Dasari A, Huang B, Shaik MN, Cesari R, Zheng X, Reynolds JM, English PA, McLachlan KR, Kern KA and LoRusso PM. A Phase I, Dose-finding Study in Patients With Advanced Solid Malignancies of the Oral Gamma-Secretase Inhibitor PF-03084014. Clin Cancer Res. 2014.
15. Von Hoff DD, LoRusso PM, Rudin CM, Reddy JC, Yauch RL, Tibes R, Weiss GJ, Borad MJ, Hann CL, Brahmer JR, Mackey HM, Lum BL, Darbonne WC, Marsters JC, Jr., de Sauvage FJ and Low JA. Inhibition of the hedgehog pathway in advanced basal-cell carcinoma. N Engl J Med. 2009; 361:1164–1172.
16. Lodish MB and Stratakis CA. Endocrine tumours in neurofibromatosis type 1, tuberous sclerosis and related syndromes. Best Pract Res Clin Endocrinol Metab. 2010; 24:439–449.
17. Yao JC, Shah MH, Ito T, Bohas CL, Wolin EM, Van Cutsem E, Hobday TJ, Okusaka T, Capdevila J, de Vries EG, Tomassetti P, Pavel ME, Hoosen S, Haas T, Lincy J, Lebwohl D, et al. Everolimus for advanced pancreatic neuroendocrine tumors. N Engl J Med. 2011; 364:514–523.
18. Nissan MH, Pratilas CA, Jones AM, Ramirez R, Won H, Liu C, Tiwari S, Kong L, Hanrahan AJ, Yao Z, Merghoub T, Ribas A, Chapman PB, Yaeger R, Taylor BS, Schultz N, et al. Loss of NF1 in cutaneous melanoma is associated with RAS activation and MEK dependence. Cancer Res. 2014; 74:2340–2350.
19. Abdulghani J, Allen JE, Dicker DT, Liu YY, Goldenberg D, Smith CD, Humphreys R and El-Deiry WS. Sorafenib sensitizes solid tumors to Apo2L/TRAIL and Apo2L/TRAIL receptor agonist antibodies by the Jak2-Stat3-Mcl1 axis. PLoS One. 2013; 8:e75414.

20. Wheler JJ, Tsimberidou AM, Falchook GS, Zinner RG, Hong DS, Fok JY, Fu S, Piha-Paul SA, Naing A and Kurzrock R. Combining erlotinib and cetuximab is associated with activity in patients with non-small cell lung cancer (including squamous cell carcinomas) and wild-type EGFR or resistant mutations. Mol Cancer Ther. 2013; 12:2167–2175.
21. Anastas JN and Moon RT. WNT signalling pathways as therapeutic targets in cancer. Nat Rev Cancer. 2013; 13:11–26.
22. Tutt A, Robson M, Garber JE, Domchek SM, Audeh MW, Weitzel JN, Friedlander M, Arun B, Loman N, Schmutzler RK, Wardley A, Mitchell G, Earl H, Wickens M and Carmichael J. Oral poly(ADP-ribose) polymerase inhibitor olaparib in patients with BRCA1 or BRCA2 mutations and advanced breast cancer: a proof-of-concept trial. Lancet. 2010; 376:235–244.
23. Ho HK, Yeo AH, Kang TS and Chua BT. Current strategies for inhibiting FGFR activities in clinical applications: opportunities, challenges and toxicological considerations. Drug Discov Today. 2014; 19:51–62.
24. Arcaro A. Targeting the insulin-like growth factor-1 receptor in human cancer. Front Pharmacol. 2013; 4:30.
25. Faivre S, Demetri G, Sargent W and Raymond E. Molecular basis for sunitinib efficacy and future clinical development. Nat Rev Drug Discov. 2007; 6:734–745.
26. Zeng Z, Sarbassov dos D, Samudio IJ, Yee KW, Munsell MF, Ellen Jackson C, Giles FJ, Sabatini DM, Andreeff M and Konopleva M. Rapamycin derivatives reduce mTORC2 signaling and inhibit AKT activation in AML. Blood. 2007; 109:3509–3512.
27. Kalous O, Conklin D, Desai AJ, O'Brien NA, Ginther C, Anderson L, Cohen DJ, Britten CD, Taylor I, Christensen JG, Slamon DJ and Finn RS. Dacomitinib (PF-00299804), an irreversible Pan-HER inhibitor, inhibits proliferation of HER2-amplified breast cancer cell lines resistant to trastuzumab and lapatinib. Mol Cancer Ther. 2012; 11:1978–1987.
28. Weston VJ, Oldreive CE, Skowronska A, Oscier DG, Pratt G, Dyer MJ, Smith G, Powell JE, Rudzki Z, Kearns P, Moss PA, Taylor AM and Stankovic T. The PARP inhibitor olaparib induces significant killing of ATM-deficient lymphoid tumor cells in vitro and in vivo. Blood. 2010; 116:4578–4587.
29. Kurzrock R, Sherman SI, Ball DW, Forastiere AA, Cohen RB, Mehra R, Pfister DG, Cohen EE, Janisch L, Nauling F, Hong DS, Ng CS, Ye L, Gagel RF, Frye J, Muller T, et al. Activity of XL184 (Cabozantinib), an oral tyrosine kinase inhibitor, in patients with medullary thyroid cancer. J Clin Oncol. 2011; 29:2660–2666.
30. Baselga J, Bradbury I, Eidtmann H, Di Cosimo S, de Azambuja E, Aura C, Gomez H, Dinh P, Fauria K, Van Dooren V, Aktan G, Goldhirsch A, Chang TW, Horvath Z, Coccia-Portugal M, Domont J, et al. Lapatinib with trastuzumab for HER2-positive early breast cancer (NeoALTTO): a randomised, open-label, multicentre, phase 3 trial. Lancet. 2012; 379:633–640.
31. Kollareddy M, Zheleva D, Dzubak P, Brahmkshatriya PS, Lepsik M and Hajduch M. Aurora kinase inhibitors: progress towards the clinic. Invest New Drugs. 2012; 30:2411–2432.
32. Young RM and Staudt LM. A new "brew" of MALT1 inhibitors. Cancer cell. 2012; 22:706–707.
33. Nagel D, Spranger S, Vincendeau M, Grau M, Raffegerst S, Kloo B, Hlahla D, Neuenschwander M, Peter von Kries J, Hadian K, Dorken B, Lenz P, Lenz G, Schendel DJ and Krappmann D. Pharmacologic inhibition of MALT1 protease by phenothiazines as a therapeutic approach for the treatment of aggressive ABC-DLBCL. Cancer cell. 2012; 22:825–837.
34. Jang S and Atkins MB. Treatment of BRAF-mutant melanoma: the role of vemurafenib and other therapies. Clin Pharmacol Ther. 2014; 95:24–31.
35. Harry BL, Eckhardt SG and Jimeno A. JAK2 inhibition for the treatment of hematologic and solid malignancies. Expert Opin Investig Drugs. 2012; 21:637–655.
36. Schmitz R, Young RM, Ceribelli M, Jhavar S, Xiao W, Zhang M, Wright G, Shaffer AL, Hodson DJ, Buras E, Liu X, Powell J, Yang Y, Xu W, Zhao H, Kohlhammer H, et al. Burkitt lymphoma pathogenesis and therapeutic targets from structural and functional genomics. Nature. 2012; 490:116–120.
37. Heidorn SJ, Milagre C, Whittaker S, Nourry A, Niculescu-Duvas I, Dhomen N, Hussain J, Reis-Filho JS, Springer CJ, Pritchard C and Marais R. Kinase-dead BRAF and oncogenic RAS cooperate to drive tumor progression through CRAF. Cell. 2010; 140:209–221.
38. Shaw AT, Kim DW, Nakagawa K, Seto T, Crino L, Ahn MJ, De Pas T, Besse B, Solomon BJ, Blackhall F, Wu YL, Thomas M, O'Byrne KJ, Moro-Sibilot D, Camidge DR, Mok T, et al. Crizotinib versus chemotherapy in advanced ALK-positive lung cancer. N Engl J Med. 2013; 368:2385–2394.
